# The Protective Role of Carbon Monoxide (CO) Produced by Heme Oxygenases and Derived from the CO-Releasing Molecule CORM-2 in the Pathogenesis of Stress-Induced Gastric Lesions: Evidence for Non-Involvement of Nitric Oxide (NO)

**DOI:** 10.3390/ijms17040442

**Published:** 2016-03-24

**Authors:** Katarzyna Magierowska, Marcin Magierowski, Marcin Surmiak, Juliusz Adamski, Agnieszka Irena Mazur-Bialy, Robert Pajdo, Zbigniew Sliwowski, Slawomir Kwiecien, Tomasz Brzozowski

**Affiliations:** 1 Department of Physiology, Jagiellonian University Medical College, 31-531 Cracow, Poland; k.jasnos@interia.pl (K.M.); marcin.surmiak@uj.edu.pl (M.S.); mppajdo@cyf-kr.edu.pl (R.P.); agazs@poczta.fm (Z.S.); skwiecien@cm-uj.krakow.pl (S.K.); mpbrzozo@cyf-kr.edu.pl (T.B.); 2 Division of Molecular Biology and Clinical Genetics, Department of Medicine, Jagiellonian University Medical College, 31-006 Cracow, Poland; 3 Department of Forensic Toxicology, Institute of Forensic Research, 31-033 Cracow, Poland; juliusz.adamski@gmail.com; 4 Department of Ergonomics and Exercise Physiology, Faculty of Health Sciences, Jagiellonian University Medical College, 31-531 Cracow, Poland; agnieszka.mazur@uj.edu.pl

**Keywords:** carbon monoxide, stress, nitric oxide, gastroprotection, gastric blood flow, heme oxygenase-1, cyclooxygenases, soluble guanylyl cyclase, hypoxia inducible factor 1α

## Abstract

Carbon monoxide (CO) produced by heme oxygenase (HO)-1 and HO-2 or released from the CO-donor, tricarbonyldichlororuthenium (II) dimer (CORM-2) causes vasodilation, with unknown efficacy against stress-induced gastric lesions. We studied whether pretreatment with CORM-2 (0.1–10 mg/kg oral gavage (i.g.)), RuCl_3_ (1 mg/kg i.g.), zinc protoporphyrin IX (ZnPP) (10 mg/kg intraperitoneally (i.p.)), hemin (1–10 mg/kg i.g.) and CORM-2 (1 mg/kg i.g.) combined with N^G^-nitro-l-arginine (l-NNA, 20 mg/kg i.p.), 1H-[1,2,4]oxadiazolo[4,3-a]quinoxalin-1-one (ODQ, 10 mg/kg i.p.), indomethacin (5 mg/kg i.p.), SC-560 (5 mg/kg i.g.), and celecoxib (10 mg/kg i.g.) affects gastric lesions following 3.5 h of water immersion and restraint stress (WRS). Gastric blood flow (GBF), the number of gastric lesions and gastric CO and nitric oxide (NO) contents, blood carboxyhemoglobin (COHb) level and the gastric expression of HO-1, HO-2, hypoxia inducible factor 1α (HIF-1α), tumor necrosis factor α (TNF-α), cyclooxygenase (COX)-2 and inducible NO synthase (iNOS) were determined. CORM-2 (1 mg/kg i.g.) and hemin (10 mg/kg i.g.) significantly decreased WRS lesions while increasing GBF, however, RuCl_3_ was ineffective. The impact of CORM-2 was reversed by ZnPP, ODQ, indomethacin, SC-560 and celecoxib, but not by l-NNA. CORM-2 decreased NO and increased HO-1 expression and CO and COHb content, downregulated HIF-1α, as well as WRS-elevated COX-2 and iNOS mRNAs. Gastroprotection by CORM-2 and HO depends upon CO’s hyperemic and anti-inflammatory properties, but is independent of NO.

## 1. Introduction

Carbon monoxide (CO), previously regarded as a metabolic waste, is now considered as a gaseous molecule exerting important signaling functions in the body [1,2]. CO can be produced by the actions of two microsomal proteins: induced by stress, heme oxygenase (HO)-1 and the constitutively expressed isoform, HO-2 [3]. Sensing gaseous molecules such as oxygen (O_2_), nitric oxide (NO) and CO are distinctive features of living organisms and are predominantly mediated by heme-based sensors [4]. Most of the actions of CO are exerted through the binding of CO to ferrous iron (Fe^2+^) and subsequent alteration of the functions of the hemoproteins [5,6]. CO competes with O_2_ for metalloproteins such as hemoglobin (Hb), myoglobin, cytochrome c oxidase and cytochrome P-450 [7,8].

Formation of carboxyhemoglobin (COHb) negatively impacts the two main functions of Hb by decreasing the O_2_ carrying capacity of blood and impairing the release of O_2_ from Hb to tissues [9]. CO has been shown to inhibit cytochrome c oxidase *in vitro*, and thus significantly inhibiting cellular respiration [10]. The interaction of CO with cytochrome P-450 in liver microsomes results in the inhibition of enzymatic activity of this protein [11]. CO, which binds to the heme moiety at the active site of soluble guanylyl cyclase (sGC), has been shown to activate sGC by about four-fold, thereby elevating intracellular levels of the second messenger molecule, cyclic guanosine 3′,5′-monophosphate (cGMP) [12]. Recently, CO has been implicated in the mechanism of gastric integrity by exhibiting sGC-activation-dependent gastroprotective effects against ethanol-induced gastric damage [13,14,15]. CO can form complexes with a reduced form of another metalloprotein, inducible NO synthase (iNOS), which is considered a pro-inflammatory marker. Activity of iNOS can be directly inhibited by CO, which is known to bind to the heme moiety of the enzyme [16,17,18]. Moreover, both cyclooxygenase (COX) isoforms, COX-1 and COX-2 are hemoproteins, which remain potential targets of CO [19,20]. The two COX isoforms are key enzymes required for the conversion of arachidonic acid to prostaglandins (PGs) [19,20]. Heme degradation subsequently begins with the generation of the ferric heme-HO complex [21]. Due to spectral similarities of heme-HO complex and both ferric myoglobin and Hb, HO seems to be another notable target of CO [22,23].

Recent studies in the field of gastrointestinal (GI) pathophysiology have consistently focused on the gastroprotective involvement of CO, NO and hydrogen sulfide (H_2_S) in various preclinical animal models of gastric mucosal lesion formation [24,25,26,27]. Moreover, Tavares *et al.* demonstrated that treatment with CO-releasing molecules (CORMs) is effective against gastric colonization by antibiotic resistant strains of *Helicobacter pylori* [28]. Restoration of delayed gastric emptying in diabetic mice to normal rates by inhalation of a low dose of CO was confirmed by Kashyap *et al.* [29]. Additionally, CO is involved in both PG-mediated stimulation of HCO_3_^−^ secretion in the duodenum [30] as well as protection against ethanol- and alendronate-induced gastric lesions [13,14,15]. CO seems to be an important factor involved in the mechanism of gastric mucosal defense, but the contribution of this gaseous molecule to gastroprotection against acute gastric lesions induced by water immersion and restraint stress (WRS) has not been explored [31,32]. WRS is a widely accepted model for studying GI erosions, which mimics the clinical outcome of stress complication in the stomach [31,32]. In particular, the role of the HO/CO system in the pathogenesis of peptic ulcer disease is still not clear. Therefore, we attempted in the present study to determine the effect of pretreatment with the CO donor, tricarbonyldichlororuthenium (II) dimer (CO-releasing molecule, CORM-2) on gastric mucosal injury induced by WRS. We examined the underlying mechanism of the potential protective action of CO with a particular focus on the ability of CORM-2 to elevate CO level in gastric mucosa and COHb concentration in whole blood. We also aimed to investigate other important factors involved in the mechanism of gastric protection such as sGC/cGMP, NO/NO synthase (NOS) and PG/COX systems by measuring both NO content in gastric tissues and changes in the mRNA expression of HO-1, HO-2, hypoxia inducible factor 1α (HIF-1α) and pro-inflammatory factors tumor necrosis factor α (TNF-α), COX-2 and iNOS in gastric mucosa following WRS.

## 2. Results

**Figure 1:**
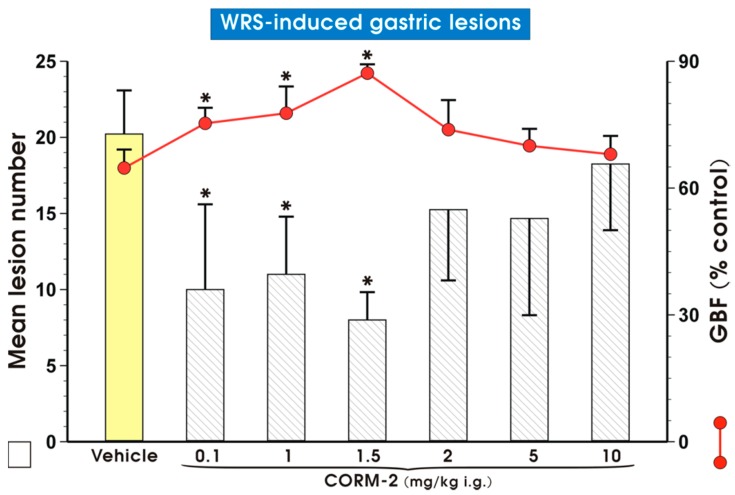
Mean lesion number and gastric blood flow (GBF) in gastric mucosa pretreated with vehicle (saline; oral gavage (i.g.)) or carbon monoxide releasing molecule 2 (CORM-2, 0.1–10 mg/kg i.g.) and compromised 30 min later by water immersion and restraint stress (WRS). Results are mean ± S.D. of 6–8 animals for each experimental group. Asterisk indicates a significant change (*p* < 0.05) as compared with vehicle.

Figure 1 shows that pretreatment with CORM-2 administered by oral gavage (i.g.) in a dose of 0.1, 1 or 1.5 mg/kg significantly reduced (*p* < 0.05) WRS-induced gastric lesions. This gastroprotective effect of CORM-2 against WRS-induced gastric lesions was accompanied by a significant increase in gastric blood flow (GBF) (*p* < 0.05). However, when CORM-2 was given in higher doses ranging from 2 to 10 mg/kg, both an increase in mean lesion number and a decrease in the GBF were observed in comparison with lower doses, which were not significantly different from those observed in vehicle-treated animals. Therefore, CORM-2 administered in the dose of 1 mg/kg, which reduced the number of WRS-induced gastric lesions by about 50% was selected and subsequently used for further studies to investigate the mechanism of CORM-2 protection against WRS-induced gastric damage.

**Figure 2:**
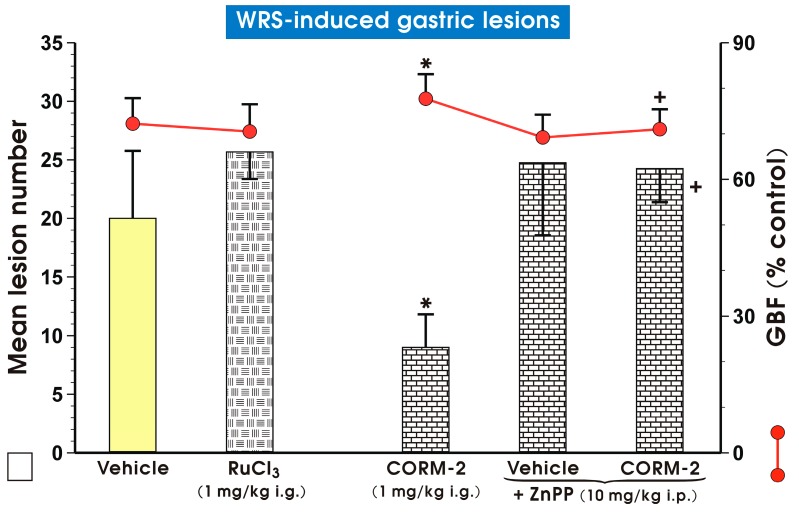
Mean lesion number and gastric blood flow (GBF) in gastric mucosa pretreated with vehicle (saline; i.g.; yellow pattern), RuCl_3_ (1 mg/kg i.g.; mosaic patttern), carbon monoxide releasing molecule 2 (CORM-2, 1 mg/kg i.g.; brick pattern) and zinc protoporphyrin IX (ZnPP, 10 mg/kg, intraperitoneally (i.p.); brick pattern) administered alone or in combination with CORM-2 and compromised 30 min later by water immersion and restraint stress (WRS). Results are mean ± S.D. of 6–8 animals for each experimental group. Asterisk indicates significant change (*p* < 0.05) as compared with vehicle (saline). Cross indicates significant change (*p* < 0.05) as compared with the group treated with CORM-2 (1 mg/kg i.g.) alone.

Figure 2 shows that RuCl_3_ (1 mg/kg i.g.), a non-CO-releasing negative control [15,30], did not significantly affect WRS-induced gastric lesions and GBF. Zinc protoporphyrin IX (ZnPP, 10 mg/kg, intraperitoneally (i.p.)), a HO-1 inhibitor [33], failed to impact the number of WRS-induced gastric lesions and GBF. However, concurrent treatment of ZnPP with CORM-2 completely reversed the CORM-2-induced decrease in WRS-induced gastric lesions and the accompanying increase in GBF (*p* < 0.05) (Figure 2).

**Figure 3:**
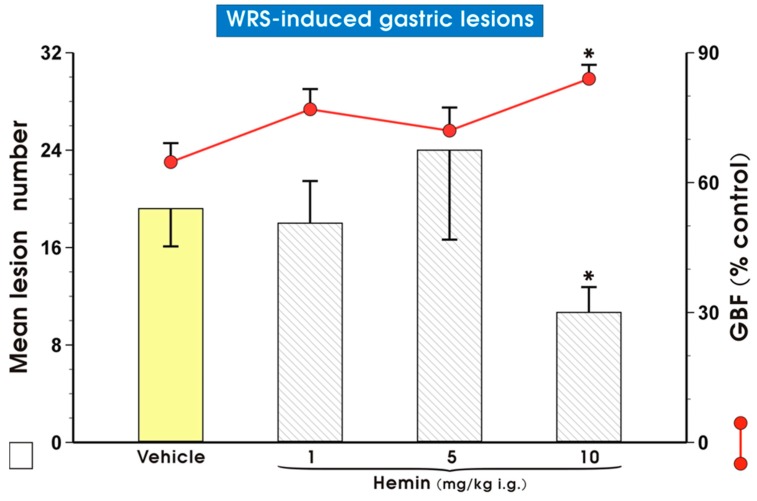
Mean lesion number and gastric blood flow (GBF) in gastric mucosa pretreated i.g. with vehicle (saline) or hemin administered in increasing doses ranging from 1 up to 10 mg/kg i.g. and exposed 30 min later to water immersion and restraint stress (WRS). Results are mean ± S.D. of 4–6 animals for each experimental group. Asterisk indicates significant change (*p* < 0.05) as compared with vehicle (saline).

Figure 3 shows the effect of pretreatment with vehicle (saline) or hemin, a HO-1 inducer [34], given i.g. in doses ranging from 1 up to 10 mg/kg on the mean lesion number and GBF in rats exposed to 3.5 h of WRS. Pretreatment with hemin administered in a dose of 1 or 5 mg/kg failed to affect the mean lesion number and GBF; however, when hemin was applied in the higher dose of 10 mg/kg, it significantly reduced (*p* < 0.05) the number of WRS-induced gastric lesions. This last effect was accompanied by a significant increase in GBF (*p* < 0.05).

**Figure 4:**
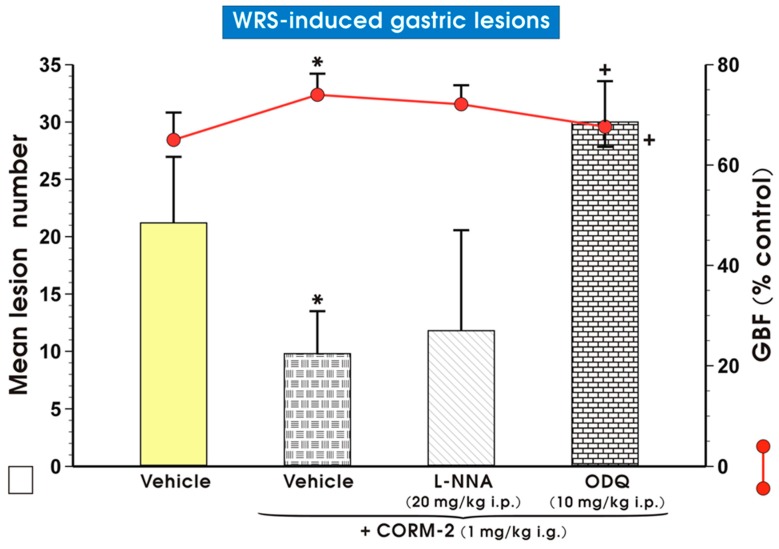
Mean lesion number and gastric blood flow (GBF) in gastric mucosa pretreated i.g. with vehicle (saline; yellow pattern), carbon monoxide releasing molecule 2 (CORM-2, 1 mg/kg i.g.) applied alone (mosaic pattern) or in combination with N^G^-nitro-l-arginine (l-NNA, 20 mg/kg i.p.; line pattern) or 1H-[1,2,4]oxadiazolo[4,3-a]quinoxalin-1-one (ODQ, 10 mg/kg i.p.; brick pattern) and followed by water immersion and restraint stress (WRS). Results are mean ± S.D. of 6–8 animals for each experimental group. Asterisk indicates significant change (*p* < 0.05) as compared with vehicle (saline). Cross indicates significant change (*p* < 0.05) as compared with the group pretreated with vehicle (saline) and administered with CORM-2 (1 mg/kg i.g.).

Figure 4 shows a simultaneous significant decrease (*p* < 0.05) in the number of WRS-induced gastric lesions and a significant increase (*p* < 0.05) in the CORM-2 (1 mg/kg i.g.) group as compared with the vehicle (saline) pretreated group. This is a similar finding to those shown in Figure 1 and Figure 2. Co-administration of CORM-2 with N^G^-nitro-l-arginine (l-NNA, 20 mg/kg i.p.), an inhibitor of NOS [35], did not cause the reduction of WRS damage elicited by this CO donor (Figure 3). Nevertheless, concomitant treatment with CO and 1H-[1,2,4]oxadiazolo[4,3-a]quinoxalin-1-one (ODQ, 10 mg/kg i.p.), which is the irreversible and highly selective inhibitor of sGC [36], reversed CORM-2-induced gastroprotection and significantly decreased GBF (*p* < 0.05) as compared with the control group treated with this CO donor alone (Figure 4).

**Figure 5:**
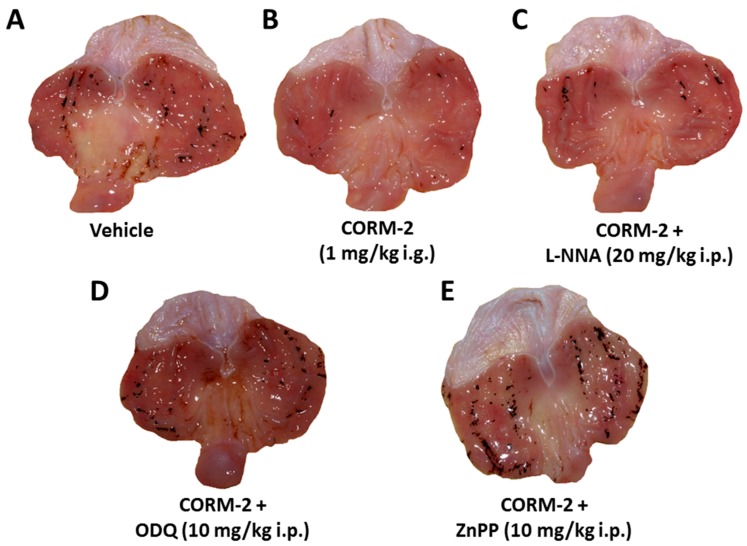
Gastric mucosa of rats exposed to water immersion and restraint stress (WRS) pretreated with vehicle (saline; i.g.) (**A**); Carbon monoxide releasing molecule 2 (CORM-2, 1 mg/kg i.g.) applied alone (**B**); or in combination with N^G^-nitro-l-arginine (l-NNA, 20 mg/kg i.p.) (**C**); 1H-[1,2,4]oxadiazolo[4,3-a]quinoxalin-1-one (ODQ, 10 mg/kg i.g.) (**D**); or zinc protoporphyrin IX (ZnPP, 10 mg/kg i.p.) (**E**). Note, the presence of hemorrhagic gastric lesions in the gastric mucosa of rats pretreated with vehicle (control) and exposed to 3.5 h of WRS (**A**). These macroscopic lesions were reduced by CORM-2 (**B**) were not affected by concurrent treatment of l-NNA (**C**) but increased in CORM-2-pretreated rats concomitantly treated with ODQ (**D**) or ZnPP (**E**).

Figure 5 shows representative photomicrographs of gastric mucosa of rats pretreated i.g. with vehicle (saline; Figure 5A) or CORM-2 (1 mg/kg i.g.; Figure 5B) following WRS. Numerous typical dot-like hemorrhagic gastric lesions were observed in vehicle-treated rats, but pretreatment with CORM-2 reduced these lesions (Figure 5A *vs.* Figure 5B). In contrast, concurrent administration of l-NNA (20 mg/kg i.p.) in combination with this CO donor failed to produce the gastric lesion reduction, which had been evoked by CORM-2 alone (Figure 5C *vs.* Figure 5B). Moreover, in the gastric mucosa of rats with concomitant administration of CORM-2 with ODQ (10 mg/kg i.p.) or ZnPP (10 mg/kg i.p.), the number of gastric lesions increased as compared with that observed in CORM-2-pretreated gastric mucosa (Figure 5D,E *vs.* Figure 5B).

**Figure 6:**
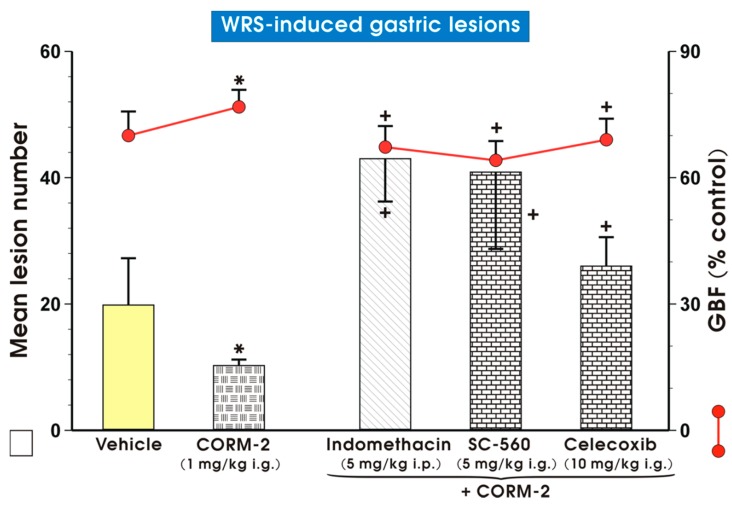
Mean lesion number and gastric blood flow (GBF) in gastric mucosa pretreated i.g. with vehicle (saline; yellow pattern), carbon monoxide releasing molecule 2 (CORM-2, 1 mg/kg i.g.) alone (mosaic pattern) or administered in combination with non-selective cyclooxygenase (COX) inhibitor, indomethacin (5 mg/kg i.p.; line pattern), selective COX-1 inhibitor, SC-560 (5 mg/kg i.g.; brick pattern) or selective COX-2 inhibitor, celecoxib (10 mg/kg i.g.; brick pattern) and 30 min later exposed to 3.5 h of water immersion and restraint stress (WRS). Results are mean ± S.D. of 6–8 animals for each experimental group. Asterisk indicates significant change (*p* < 0.05) as compared with vehicle (saline) controls. Cross indicates significant change (*p* < 0.05) as compared with the group treated with CORM-2 (1 mg/kg i.g.) alone.

Figure 6 shows that CORM-2 (1 mg/kg i.g.) caused a significant reduction in WRS-lesion number as well as an increase in GBF, similarly as demonstrated in Figure 1, Figure 2 and Figure 4. Administration of indomethacin (5 mg/kg i.p.), SC-560 (5 mg/kg i.g.) or celecoxib (10 mg/kg i.g.), non-selective and selective COX-1 or COX-2 inhibitors [37], respectively, prior to CORM-2 (1 mg/kg i.g.) significantly increased (*p* < 0.05) WRS-induced lesions and decreased GBF compared to respective values obtained in rats pretreated with this CO donor alone (Figure 6).

**Figure 7:**
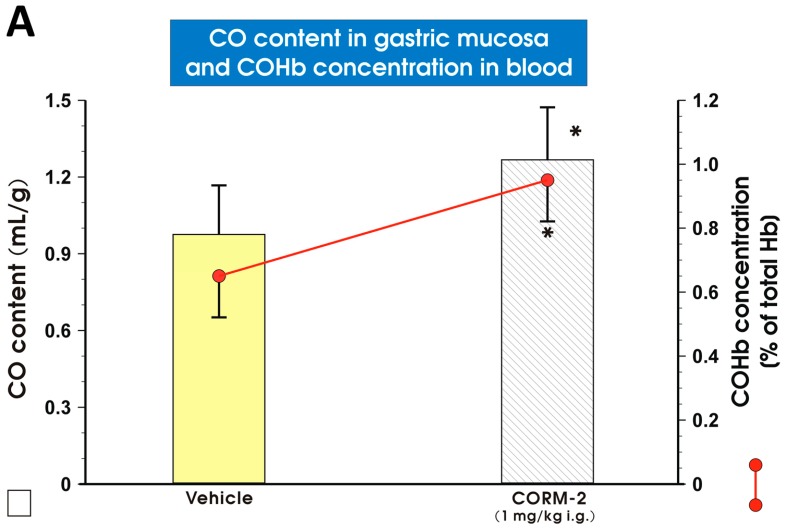

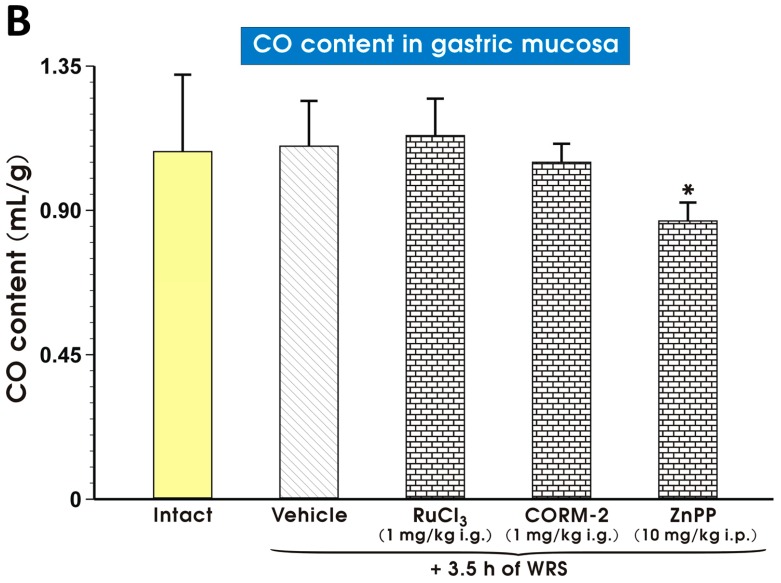
The gastric mucosal content of carbon monoxide (CO) and carboxyhemoglobin (COHb) concentration in rats treated i.g. with vehicle (saline; yellow pattern) and CORM-2 (1 mg/kg; line pattern) (**A**); and the gastric mucosal CO content in intact rats (yellow pattern) and in rats pretreated with vehicle (saline; i.g.; line pattern), RuCl_3_ (1 mg/kg i.g.; brick pattern), CO releasing molecule 2 (CORM-2 ,1 mg/kg i.g.; brick pattern) and zinc protoporphyrin IX (ZnPP, 10 mg/kg i.p.; brick pattern) 30 min before the exposure to 3.5 h of water immersion and restraint stress (WRS) (**B**). Results are mean ± S.D. of 4–5 animals for each experimental group. Asterisk indicates significant change (*p* < 0.05) as compared with vehicle-treated groups.

Figure 7A shows the gastric mucosal CO content and COHb concentration in blood of rats pretreated with vehicle (saline) or CORM-2 (1 mg/kg i.g.). The CO content in the gastric mucosa and COHb concentration in blood were significantly increased (*p* < 0.05) after CORM-2 (1 mg/kg i.g.) administration, as compared with vehicle-treated animals (Figure 7A). The content of CO in the gastric mucosa pretreated with vehicle (saline), RuCl_3_, CORM-2 or ZnPP and exposed 30 min later by WRS is presented in Figure 7B. In rats pretreated with vehicle (saline), CO content was not significantly affected as compared with intact animals (Figure 7B). The gastric mucosal content of CO in rats pretreated with RuCl_3_ (1 mg/kg i.g.) or CORM-2 (1 mg/kg i.g.) 30 min before exposure to WRS failed to show a significant difference as compared with the vehicle-treated group (Figure 7B). However, ZnPP (10 mg/kg i.p.) significantly decreased (*p* < 0.05) the CO gastric mucosal content in rats compromised by WRS as compared with the vehicle-treated group (Figure 7B).

**Figure 8:**
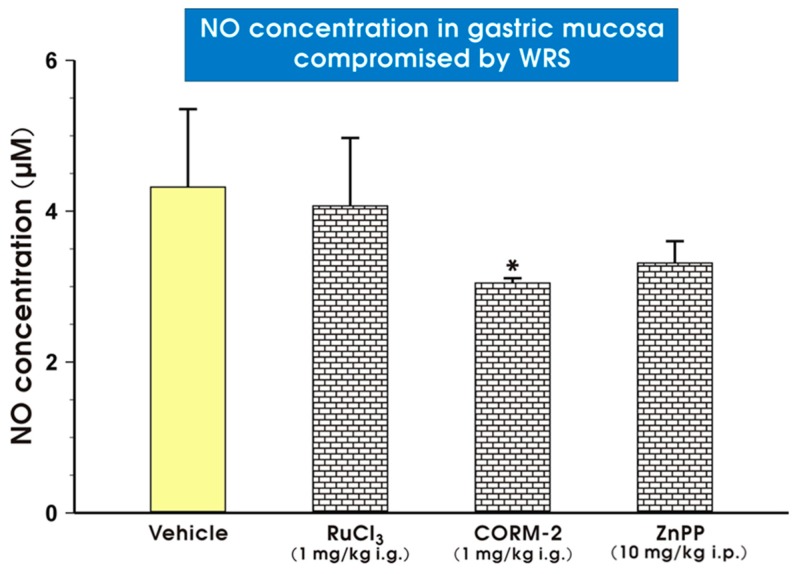
The gastric mucosal nitric oxide (NO) content in rats pretreated i.g. with vehicle (saline; yellow pattern), RuCl_3_ (1 mg/kg; brick pattern), carbon monoxide releasing molecule 2 (CORM-2, 1 mg/kg; brick pattern) or zinc protoporphyrin IX (ZnPP, 10 mg/kg i.p.; brick pattern) and exposed 30 min later to 3.5 h of water immersion and restraint stress (WRS). Results are mean ± S.D. of 4–5 animals in each experimental group. Asterisk indicates significant change (*p* < 0.05) as compared with vehicle-treated control group.

Figure 8 shows that CORM-2 administered in a dose of 1 mg/kg i.g. significantly decreased (*p* < 0.05) NO concentrations in gastric mucosa of rats exposed to WRS as compared with vehicle-treated rats. The gastric mucosal NO content in intact animals was 3.2 ± 0.9 µM and significantly elevated (*p* < 0.05) to 4.4 ± 1.2 µM in the gastric mucosa of rats pretreated with vehicle (saline) and exposed to WRS. Pretreatment with RuCl_3_ (1 mg/kg i.g.) failed to impact NO content in the gastric mucosa compromised by WRS as compared with the vehicle-treated group, while ZnPP administered in a dose of 10 mg/kg i.p. did not significantly change gastric NO content as compared with vehicle treated rats (Figure 8).

**Figure 9:**
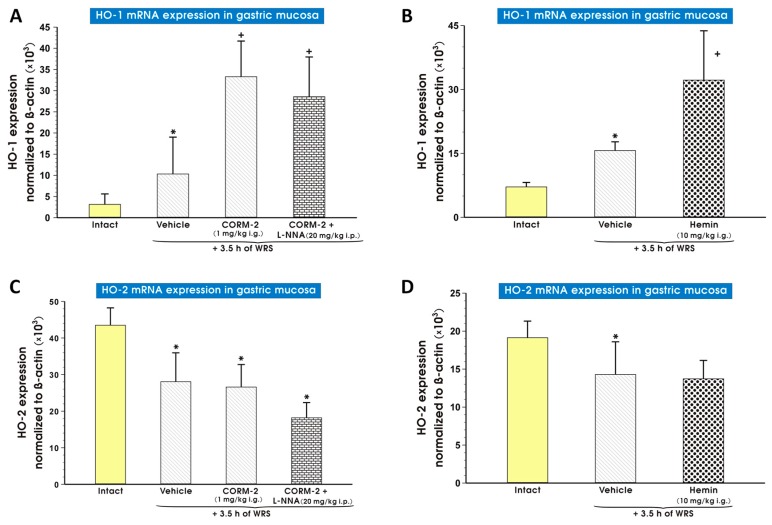
Expression levels of mRNA for heme oxygenase (HO)-1 (**A**,**B**) and HO-2 (**C**,**D**) in the gastric mucosa of intact rats (yellow pattern) or those pretreated with vehicle (saline; i.g.; line pattern), hemin (10 mg/kg i.g.; dot pattern) or carbon monoxide releasing molecule 2 (CORM-2, 1 mg/kg i.g.) alone (line pattern) or administered in combination with N^G^-nitro-l-arginine (l-NNA, 20 mg/kg i.p.; brick pattern) and exposed to 3.5 h of water immersion and restraint stress (WRS). Results are expressed as mRNA expression of HO-1 and HO-2 normalized to β-actin. Results are mean ± S.D. of 6–8 determinations per group. Asterisk indicates a significant change (*p* < 0.05) as compared with the respective values in intact gastric mucosa. Cross indicates significant change (*p* < 0.05) as compared with the values obtained for the vehicle-treated group.

Figure 9 shows mRNA expression levels of HO-1 (Figure 9A,B) and HO-2 (Figure 9C,D) in the gastric mucosa of both intact rats and those pretreated with saline (vehicle), hemin (10 mg/kg i.g.; Figure 9B), or CORM-2 (1 mg/kg i.g.) alone or combined with l-NNA (20 mg/kg i.p.) and subsequently exposed to WRS (Figure 9A). In vehicle-treated rats exposed to WRS, a significant increase (*p* < 0.05) in gastric mucosal mRNA expression of HO-1 was observed as compared with intact rats (Figure 9A,B). Pretreatment with hemin (10 mg/kg i.g.) or CORM-2 (1 mg/kg i.g.) significantly increased (*p* < 0.05) HO-1 mRNA expression over that of vehicle-treated rats exposed to WRS. This effect of the CO donor was not significantly affected by l-NNA (Figure 9A,B). Expression of HO-2 mRNA was significantly decreased (*p* < 0.05) in vehicle-pretreated rats who were exposed to WRS as compared with intact animals (Figure 9C,D). CORM-2 (1 mg/kg i.g.) or hemin administered in a dose of 10 mg/kg i.g. applied alone or combined with l-NNA (20 mg/kg i.p.) did not change the downregulation of HO-2 mRNA expression observed in vehicle-control rats exposed to WRS (Figure 9C,D).

**Figure 10:**
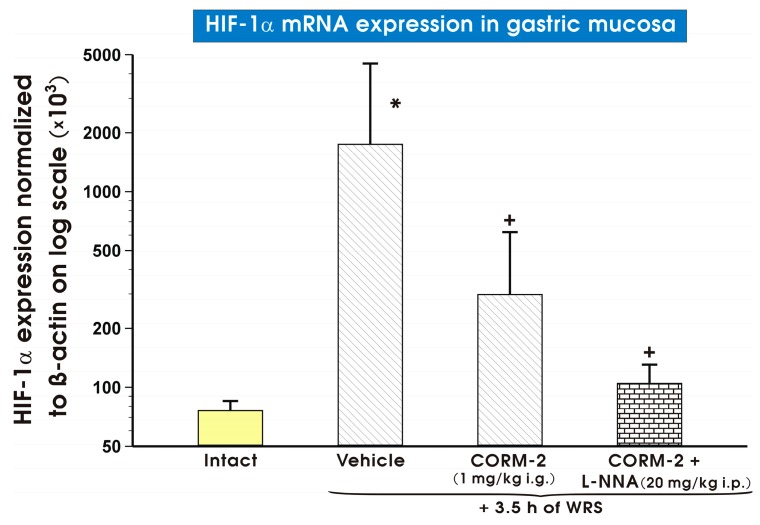
Expression levels of hypoxia inducible factor 1α (HIF-1α) mRNA in the gastric mucosa of intact rats (yellow pattern) or those pretreated with vehicle (saline; i.g.; line pattern), carbon monoxide releasing molecule 2 (CORM-2, 1 mg/kg i.g.; line pattern) alone or in combination with N^G^-nitro-l-arginine (l-NNA, 20 mg/kg i.p.; brick pattern) and exposed to 3.5 h of water immersion and restraint stress (WRS). Results are expressed as HIF-1α mRNA expression normalized to β-actin. Results are mean ± S.D. of 6–8 determinations per group. Asterisk indicates a significant change (*p* < 0.05) as compared with the respective values in intact gastric mucosa. Cross indicates a significant change (*p* < 0.05) as compared with the values obtained vehicle-treated group.

Figure 10 shows mRNA expression levels of HIF-1α in the gastric mucosa of both intact rats and those pretreated with either vehicle (saline) or CORM-2 (1 mg/kg i.g.), which were applied with or without combination with l-NNA (20 mg/kg i.p.) and subsequently exposed to 3.5 h of WRS. In vehicle-treated gastric mucosa, a significant increase (*p* < 0.05) in gastric mucosal HIF-1α mRNA expression was observed as compared with intact rats (Figure 10). CORM-2, applied either alone or administered in combination with l-NNA significantly decreased (*p* < 0.05) HIF-1α mRNA expression as compared with the vehicle-control group (Figure 10).

**Figure 11:**
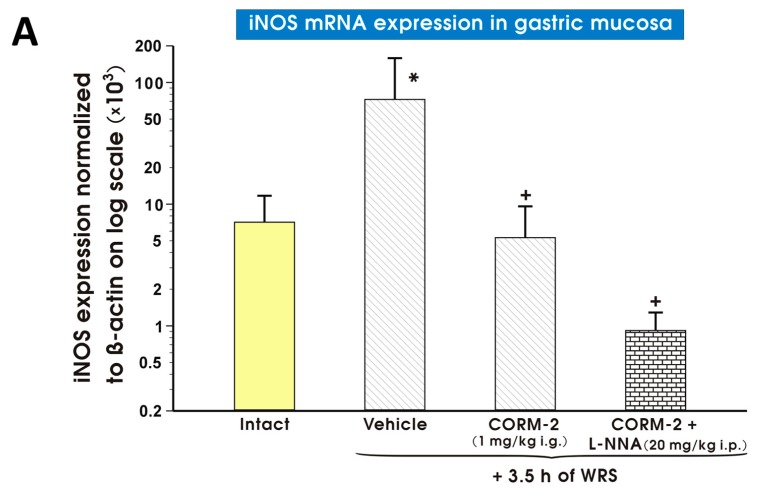

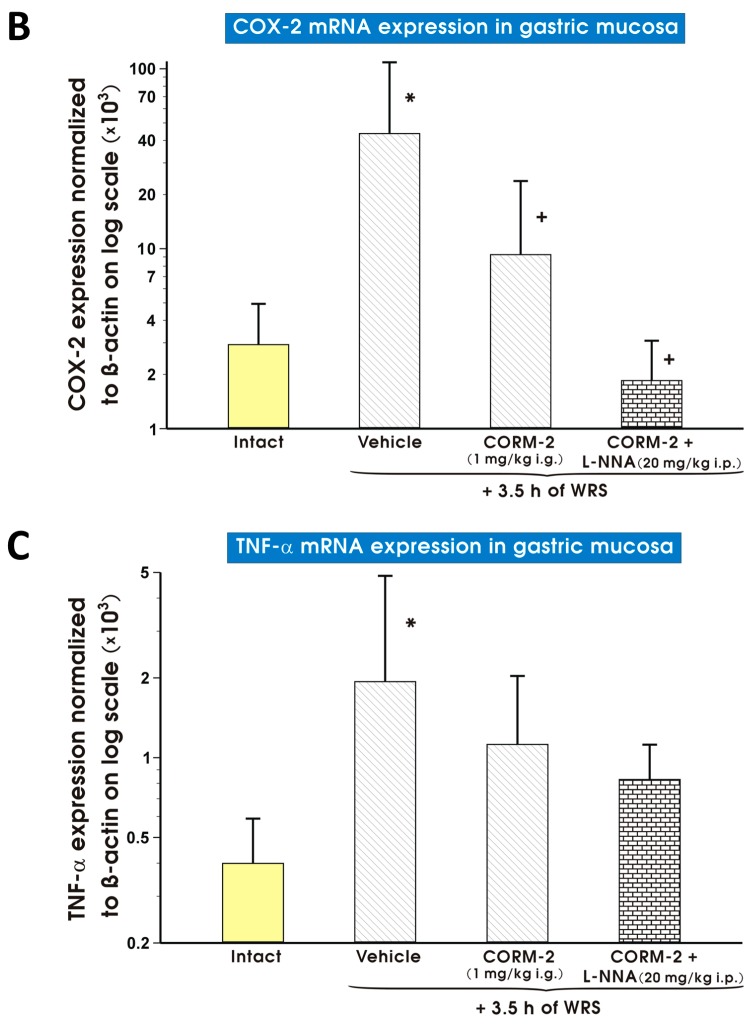
Expression levels of mRNA for inducible nitric oxide synthase (iNOS) (**A**); cyclooxygenase 2 (COX-2) (**B**); or tumor necrosis factor α (TNF-α) (**C**) in the gastric mucosa of intact rats (yellow pattern) or those pretreated with vehicle (saline; i.g.; line pattern), carbon monoxide releasing molecule 2 (CORM-2, 1 mg/kg i.g.; line pattern) alone or in combination with N^G^-nitro-l-arginine (l-NNA, 20 mg/kg i.p.; brick pattern) in rats 30 min later exposed to 3.5 h of water immersion and restraint stress (WRS). Results are expressed as mRNA expression of iNOS, COX-2 and TNF-α normalized to β-actin. Results are mean ± S.D. of 6–8 determinations per group. Asterisk indicates a significant change (*p* < 0.05) as compared with the respective values in intact gastric mucosa. Cross indicates significant change (*p* < 0.05) as compared with the values obtained vehicle-treated group.

As shown in Figure 11A–C, the gastric mucosal mRNA expression of iNOS (Figure 11A), COX-2 (Figure 11B) or TNF-α (Figure 11C) was significantly augmented in vehicle-treated control gastric mucosa as compared with those detected in intact rats. CORM-2 (1 mg/kg i.g.) administered alone or in combination with l-NNA (20 mg/kg i.p.) significantly decreased (*p* < 0.05) mRNA expression for iNOS (Figure 11A) and COX-2 (Figure 11B) as compared with vehicle-treated gastric mucosa (Figure 11A,B). Pretreatment with CORM-2 (1 mg/kg i.g.) did not significantly influence the rise in mRNA expression for TNF-α observed in vehicle-treated rats exposed to WRS (Figure 11C).

## 3. Discussion

Exposure of rats to acute stress is known to cause hemorrhagic gastric lesions in the stomach and mimics the clinical appearance of stress-induced peptic ulcers observed clinically in humans who suffer from burns, cold and life-threatening conditions [31,32,38]. Gastric hemorrhagic erosions following stress are sometimes called “stress ulcerations”. They have been attributed to hyperacidity resulting from changes in gastric acid secretion, hypermotility and increased permeability of the gastric mucosa to H^+^ ions involving deterioration of the microcirculation [39,40,41].

We observed that endogenous CO, produced due to the activity of HO or released from an exogenous chemical donor, CORM-2, administered i.g. in low doses ranging from 0.1 to 1.5 mg/kg, can attenuate the formation of acute stress-induced gastric lesions. This protective effect was accompanied by an increase in GBF, which in turn suggests a crucial role for the rise in the gastric microcirculation in this protection. Interestingly, we found that higher doses of CORM-2 from 2 up to 10 mg/kg were ineffective in gastroprotection against stress lesions. This finding likely suggests that increased amount of CORM-2-derived CO negatively impacted gastric cell respiration due to increased bioavailability of CO and COHb content in gastric mucosa and blood, respectively. Our previous study [15] revealed that CORM-2 dose-dependently increased COHb concentration in blood and CO content in gastric mucosa. Moreover, CORM-2 applied i.g. in a dose of 100 mg/kg exacerbated ethanol-induced gastric lesions and markedly decreased GBF. These earlier findings suggested a strict dependence of this gastroprotection and an increase in GBF on the dose of this CO donor [15]. Altogether, using the stress model of gastric injury, we confirmed that the beneficial or noxious action of CORM-2 released CO depends upon its administered dosage. The relevant dosage may in turn vary depending on the type of injury, such in case of hemorrhagic ethanol damage or stress-induced mucosal erosions. Furthermore, we found that mRNA expression for inducible HO-1 was upregulated and pretreatment with CORM-2 (1 mg/kg i.g.) further enhanced this effect in gastric mucosa compromised by WRS. Similarly to our results, both Morsy *et al.* and Ibrahim *et al.* reported an increase in gastric HO-1 expression in response to cold stress as determined by immunohistochemistry or enzyme-linked immunosorbent assay (ELISA), respectively [42,43]. We observed that the HO-1 inducer, hemin, in the highest dose (10 mg/kg i.g.), reduced WRS-induced gastric lesions while simultaneously increasing GBF and HO-1 mRNA expression. These findings confirm our previous observation [15] that CORM-2 and hemin administered in the dose of 5 mg/kg i.g., prevented gastric lesions induced by a corrosive agent, such as ethanol via enhancement of GBF and upregulation for HO-1 mRNA expression by CORM-2. Interestingly, mRNA expression for HO-2, which is expressed constitutively in parietal and gastrin cells of the stomach [44], was downregulated in gastric mucosa that was compromised by WRS in the current study. However, CORM-2 (1 mg/kg i.g.) or hemin, administered in protective dose of 10 mg/kg did not produce the same results. This finding implies that the gastroprotective mechanism of CORM-2 against WRS damage involves the upregulation of HO-1 but not the HO-2, and that CO derived from HO-1 can compensate for the decrease in HO-2 expression in stressed gastric mucosa. Our previous study had revealed that protein expression for HO-2 was unchanged in gastric mucosa compromised by ethanol but CORM-2 administered in gastroprotective dose of 5 mg/kg i.g. decreased the protein expression level of this enzyme [15].

We previously attributed the gastroprotective effect of CORM-2 against ethanol-induced gastric damage to the release of CO since COHb levels and the content of this gaseous mediator in gastric mucosa were both increased after i.g. administration of CORM-2 in a dose of 5 mg/kg [15]. Similarly, we have now observed that COHb levels and CO content in the gastric mucosa were increased after CORM-2 application in a dose of 1 mg/kg. This increase conferred gastric mucosal protection against stress-induced lesions accompanied by an increase in GBF. Moreover, we have demonstrated that the application of RuCl_3_, employed as a non-CO-releasing negative control to CORM-2, which did not confer protection against ethanol damage [15], failed to influence on the stress-induced gastric lesions and accompanying alterations in GBF. This observation is corroborative with the observation by Takeuchi *et al.* [45] who revealed that the beneficial effect of CORM-2 on protective alkaline bicarbonate secretion is solely due to CO generated from this compound because RuCl_3_ has been used by this group of investigators as a negative control to CORM-2 and had no effect on this secretion. It is therefore assumed that only CO released from CORM-2, but not ruthenium itself, is responsible for the gastroprotective and hyperemic effects observed in our present study.

Additionally, administration of ZnPP, a non-selective HO inhibitor, decreased CO content in gastric mucosa compromised by WRS, and also reversed the gastroprotective effect of CORM-2 when concomitantly administered with this CO donor. Therefore, we concluded that CO produced from heme either by the HO-1/HO-2 pathway or released from CORM-2 exerts gastroprotective activity in the gastric mucosa against stress-induced injury. ZnPP has been previously reported to amplify ethanol- and hydrochloric acid-induced gastric damage [13,46], but in our present study ZnPP failed to affect stress-induced gastric damage by itself. ZnPP did abolish the CORM-2-induced gastroprotection against these lesions and also decreased CO content in gastric mucosa (Figure 7B) possibly via inhibition of HO activity. Therefore, we concluded that CORM-2-induced-gastroprotection could be mediated by both CO release and induction of HO-1 caused by this agent. The mechanism of gastroprotection of CO produced by both CORM-2 and HO-1 should be further examined. Our finding support the notion that CORM-2 in the protective dose used was not sufficient to counteract the deficit in CO content resulting from the inhibition of HO by ZnPP. However, our observations using ZnPP in the stress-injured rat stomach seem to be at variance with those by Ibrahim *et al.* [42] who reported that 10 days of ZnPP administration reduced stress-induced gastric lesions. They had also concluded that the protective effect of ZnPP against gastric ulcers induced by cold stress might be related to an inhibition of gastric acidity and the availability of zinc from this HO inhibitor. These conclusions were made despite the apparent inhibition of endogenous CO by ZnPP, while zinc has been shown to exert gastroprotective and anti-ulcer effects. This discrepancy between our results and their observation [42] may be due to different experimental conditions. Such difference include the combined method of stress induction with the use of pylorus ligating for assessment of gastric acid secretion and exposure to cold stress along with an experimental design, which included chronic (10 days) administration of this HO inhibitor [42] *vs.* the application of ZnPP in a single dose prior to stress exposure in our present work.

Interestingly, our present study revealed that CO protection against stress-induced acute gastric injury is independent of NO since the blockade of NOS by the non-selective inhibitor, l-NNA [35], failed to influence the protective effect of HO-derived and CORM-2-released CO. Moreover, we demonstrated that CORM-2 administered in a protective dose of 1 mg/kg downregulated gastric mucosal mRNA expression for iNOS in rats exposed to WRS. The administration of CORM-2 also decreased NO content in gastric mucosa compromised by WRS. Inhibition of iNOS expression by CO has been previously reported in LPS-stimulated macrophages by Srisook *et al.* and Sawle *et al.* [17,47] who findings are partially in line with our observations. Therefore, it is reasonable to assume that CO-induced gastroprotection against stress injury does not involve the contribution of NO activity in CORM-2 protection against stress-induced gastric lesions. As shown before [15], this assumption could not explain the mediatory role of NO in CORM-2-induced gastroprotection against ethanol injury. This notion is supported by our recently published findings [15] that inhibition of NOS by l-NNA reduced gastroprotection of CORM-2 against gastric damage induced by ethanol. This controversy whether NO could mediate CO-induced protection should be further studied but we think that this discrepancy may be due to the difference in the experimental models of gastric damage induced by topically applied necrotizing agent ethanol *vs.* pleiotropic stress lesions induced by many non-topical and local factors including ischemia, hypoxia and/or initiation of oxidative processes [38,48,49].

We have demonstrated that sGC inhibitor ODQ completely reversed CORM-2-induced gastroprotection and an increase in GBF in the gastric mucosa compromised by WRS. This observation suggests that CO-induced gastroprotection against stress injury and regulation of gastric microcirculation may involve the activity of sGC/cGMP signaling pathway. This supports the previous findings [14,15] that sGC/cGMP pathway is involved in the protective effect of CO in alendronate- and ethanol-induced gastric injury.

It has been shown that endogenous PGs produced by COX-1 and COX-2 expression and activity are important components of gastric mucosal barrier [50,51,52]. These factors were proposed as keynote mediators of gastroprotection involved in the maintenance of gastric mucosal integrity [50,51,52]. Thus, we hypothesized that PGs considered as classic cytoprotective agents [52], can mediate the CO-induced gastroprotection and an increase in GBF in the rat stomach under stress conditions. Herein, we demonstrated that non-selective (indomethacin) and selective COX-1 and COX-2 inhibitors (SC-560 and celecoxib, respectively) reversed gastroprotective effect of CORM-2 and decreased GBF elevated by this CO donor in the gastric mucosa compromised by WRS. This indicates that CO released from CORM-2 can interact with classic gastroprotective arachidonate metabolites PGs in the mechanism of gastroprotection and accompanying gastric mucosal hyperemia.

This study revealed that CO-mediated gastroprotection against stress-induced gastric lesions involves the reduction of hypoxia documented by the decrease in mRNA expression for HIF-1α. The pathophysiologic mechanism of the stress-induced gastric damage involves generation of reactive oxygen species (ROS) [41], a well-recognized markers of the mucosal oxidative stress associated with regulation of HIF-1α expression and lipid peroxidation in hypoxic gastric mucosa [53]. Indeed, Gomes *et al.* [13] revealed that administration of CO-releasing dimanganese decacarbonyl (DMDC) or induction of HO-1 by hemin decreased malonylodialdehyde (MDA) formation and increased reduced glutathione (GSH) concentration in the gastric mucosa with ethanol-induced gastric damage. Moreover, Costa *et al.* [14] demonstrated that DMDC decreased MDA concentrations, and also increased the levels of GSH in gastric mucosa compromised by alendronate. Although not studied in our present study, it seems likely that CORM-2 releasing CO-mediated reduction in ROS formation and lipid peroxidation could contribute to the beneficial protective effect of this gaseous molecule observed in gastric mucosa compromised by stress.

Moreover, we demonstrated that CO released from CORM-2 reduced inflammation since pretreatment with CORM-2 (1 mg/kg i.g.) decreased mRNA expression for pro-inflammatory isoforms COX-2 and iNOS in the gastric mucosa compromised by WRS. Our results are in agreement with previously published reports regarding diminished iNOS [15,54,55,56,57] or COX-2 [15,52] expression in the various disorders of upper GI tract. However, pretreatment with CORM-2 did not affect mRNA expression for pro-inflammatory cytokine TNF-α, which is upregulated in the gastric mucosa exposed to WRS. This observation remains in contrast to decreased TNF-α concentration in the gastric mucosa of rats pretreated with CO donor and exposed to alendronate [14]. Again, this lack of influence of CORM-2 on TNF-α expression could be due to different models of gastric injury alendronate *vs.* stress and the duration time between the beginning and termination of the experiment in studies with alendronate [14]. Interestingly, the inhibition of NOS by l-NNA had no effect whatsoever on anti-hypoxic and anti-inflammatory activity of CO released from CORM-2 in the gastric mucosa exposed to WRS because CORM-2-induced downregulation of local mRNA expression for HIF-1α, COX-2 and iNOS was not modified by concurrent treatment with l-NNA. This observation supports our conclusion that NO is not involved in the beneficial protective and hyperemic activity of CO against stress lesions, which mainly depends upon the activation of the HO-1 pathway, sGC/cGMP system and endogenous PGs by this vasoactive mediator.

## 4. Materials and Methods

### 4.1. Animals, Experimental Design, Chemicals and Drugs Treatment

Male Wistar rats with average weight 220–300 g were used in the experiments. Animals were deprived of food for 24 h with free access to tap water before any application. All procedures were approved by the Institutional Animal Care and Use Committee of Jagiellonian University Medical College in Cracow and in accordance with Helsinki Declaration (Application No.: 151/VIII/2012; Decission No.: 137/2012; Date: 19 September 2012).

**Scheme 1:**
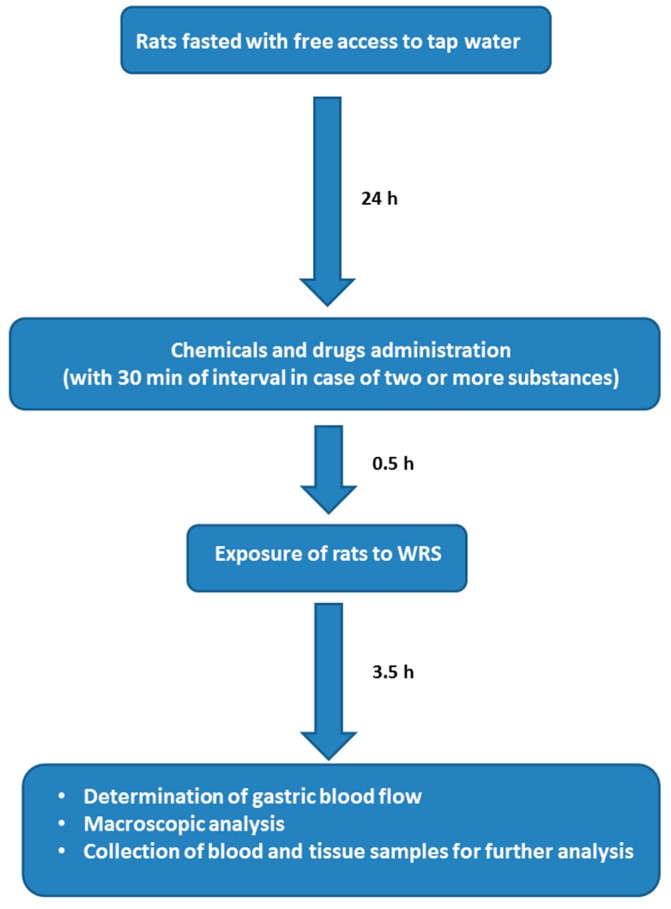
Experimental protocol and sequence of performed procedures. WRS: water immersion and restraint stress.

The experimental protocol and the sequence of procedures are depicted in Scheme 1.

To induce gastric lesions by stress, rats were immobilized in individual Bolman’s cages and immersed in the cold water (21 °C) for 3.5 h to the level of xiphoid cartilage as originally described by Takagi *et al.* and in our previous studies [51,58].

Chemicals and drugs were administered i.p. or i.g. using an orogastric tube as previously reported [59].

Animals were randomly placed into six groups (A, B, C, D, E and F; 6–8 animals each) that were pretreated 30 min before exposure to WRS with appropriate chemicals and drugs.

In Group A, rats were treated with: (1) vehicle (saline; 1 mL/rat i.g.) or (2) CORM-2 (Sigma-Aldrich, Schnelldorf, Germany) administered i.g. in doses ranging from 0.1 up to 10 mg/kg.

In Group B, rats were pretreated with: (1) vehicle (saline; i.g.); (2) RuCl_3_ (1 mg/kg i.g., Sigma-Aldrich) as a negative control to CORM-2 [15,30,45]; (3) CORM-2 applied i.g. in a dose of 1 mg/kg which in our preliminary studies reduced stress-induced gastric lesions by about 50% (series A); and (4) ZnPP (10 mg/kg i.p.), the HOs inhibitor [33], administered alone or in combination with CORM-2.

In Group C, rats were pretreated with hemin, HO-1 inducer [34], applied i.g., in doses ranging from 1 to 10 mg/kg.

Rats of Group D were pretreated with: (1) l-NNA (20 mg/kg i.p.), non-selective NOS inhibitor [35]; or (2) ODQ (10 mg/kg i.p., Sigma-Aldrich), a sGC inhibitor [36], 30 min before CORM-2 (1 mg/kg i.g.) or vehicle (saline; i.g.) application.

In Group E, animals were pretreated with: (1) vehicle (saline; i.g.); or (2) CORM-2 (1 mg/kg i.g.) alone or in combination with the non-selective COX inhibitor, indomethacin (5 mg/kg i.p., Sigma-Aldrich) or the selective COX-1 inhibitor, SC-560 (5 mg/kg i.g., Cayman Chemical, Ann Arbor, MI, USA), or celecoxib (10 mg/kg i.g., Pfizer, Illertissen, Germany), a selective COX-2 inhibitor [37].

In order to determine the CO level in gastric mucosa and COHb concentrations in the blood samples, rats of separate Group F were not exposed to WRS, but were treated i.g. with 1 mL of saline or CORM-2 applied i.g. in a dose of 1 mg/kg.

### 4.2. Determination of Gastric Blood Flow (GBF) and Gastric Lesion Number

At the termination of 3.5 h of WRS (Groups A, B, C, D, E) or 30 min after i.g., saline or CORM-2 (1 mg/kg i.g.) application (Group F), rats were kept under pentobarbital (60 mg/kg i.p.) anesthesia in order to open the stomachs for the GBF measurement using H_2_-gas clearance technique as described previously [60]. Briefly, the GBF was assessed in the oxyntic part of the gastric mucosa not having stress-induced mucosal lesions. Average values of three determinations were expressed as a percentage of vehicle-treated gastric mucosa. Gastric lesion number in each rat stomach from Groups A, B, C, D and E was determined blindly using computerized planimetry (Morphomat, Carl Zeiss, Berlin, Germany) [37,61]. Gastric mucosal biopsies (about 500 mg each) were taken for determinations of CO content and the remaining part of stomach was scraped off on ice, snap-frozen in liquid nitrogen and stored at −80 °C until further analysis [15]. The blood samples (about 3 mL each) were taken from the *vena cava* for the measurement of COHb concentrations [15].

### 4.3. Measurement of Carbon Monoxide (CO) Content in Gastric Mucosa and Blood Carboxyhemoglobin (COHb) Concentration Using Gas Chromatography (GC)

CO concentrations in the gastric mucosa and COHb levels in blood were determined using GC based on method described previously [15,62]. Briefly, the method is based on CO release from Hb due to the change in the oxidation of Fe ion from +2 to +3 localized in the porphyrin ring center and the catalytical conversion of CO to CH_4_, which was quantified by the use of flame ionization detector (FID).

The detector response was expressed as CO volume using calibration curve assembled by adding standards containing 0.0, 0.5, 1.0, 2.0, 5.0, 10.0, 20.0 and 50.0 mL of CO. Due to differences in Hb level in particular blood samples and low values of COHb concentrations, it was necessary to construct the calibration curve separately for each blood investigated, as described in details previously [62]. Briefly, standard solutions containing 100% of COHb were obtained after complete saturation of each blood sample with CO and subsequently diluted with water to obtain solutions containing 10.0%, 7.5%, 5.0%, 2.5% and 0.0% of COHb. Standards for each calibration level were prepared in triplicate. Chromatographic separation was performed in isothermal mode and appropriate instrumental parameters of the headspace GC-FID method using column packed with molecular sieves [15].

### 4.4. Determination of Nitric Oxide (NO) Content in Gastric Mucosa

Tissue biopsies stored at −80 °C were homogenized with a cold phosphate-buffered saline (Polfa, Kutno, Poland), centrifuged (3000 rpm, 15 min, 4 °C) and the supernatant was used for further analysis. Before nitrite (NO_2_)^−^ and nitrate (NO_3_)^−^ determination, samples were normalized for protein concentration using the standard Bradford reaction (Sigma-Aldrich). NO levels were quantified using an Arrowstraight™ Nitric Oxide measurement system (Lazar Research Laboratories, Los Angeles, CA, USA), which contained micro ion selective electrodes for independent measures of both nitrite and nitrate in 100 µL of sample. As a standard, the sodium nitrite and sodium nitrate were used (Sigma-Aldrich, Natick, MA, USA; for both). All procedures were conducted according to the manufacturer’s protocol. Results were presented as a total NO (µM) calculated by summing the concentration values of (NO_2_)^−^ and (NO_3_)^−^.

### 4.5. Determination of mRNA Expression Levels for Heme Oxygenase (HO)-1, HO-2, Hypoxia Inducible Factor 1α (HIF-1α), Cyclooxygenase (COX)-2, Inducible NO Synthase (iNOS) and Tumor Necrosis Factor α (TNF-α) in Gastric Mucosa

Expression levels of mRNA in gastric mucosa were determined by real-time polymerase chain reaction (qPCR) as described previously [15]. Briefly, RNA was isolated from gastric mucosal biopsies using GeneMATRIX Universal RNA Purification Kit (EURx, Gdansk, Poland). High-Capacity cDNA Reverse Transcription Kit (Thermo Fisher Scientific, Cambridge, MA, USA) was used to perform reversed transcription to cDNA. Expression levels of HO-1, HO-2, HIF-1α, TNF-α, COX-2, iNOS and β-actin as an internal control were determined using specific primers presented in Table 1, SG qPCR Master Mix (2×) including SYBR-Green (EURx, Gdansk, Poland) and thermal cycler 7900HT Fast Real-Time PCR System (Thermo Fisher Scientific). Results were analyzed using the 2^−Δ*C*t^ method [63].

### 4.6. Statistical Analysis

Results are expressed as mean ± S.D. Statistical comparison was performed by Mann–Whitney *U*-test or Student’s *t*-test. Analysis of variance (ANOVA) with Tukey *post-hoc* or Kruskal–Wallis with Dunns *post-hoc* test where used where appropriate. *p* < 0.05 was considered significant.

## 5. Conclusions

We conclude that CO produced endogenously via heme degradation or CO released from a chemical donor, CORM-2, can confer protection against stress-induced mucosal lesions. The mechanism of CO gastroprotection may involve the regulation of gastric microcirculation due to activation of the sGC/GMP system as well as anti-hypoxic and anti-inflammatory actions of this gaseous molecule. Moreover, we have demonstrated that CO gastroprotection against stress-induced gastric lesions could be mediated by endogenous PGs, the cytoprotective products of arachidonate metabolism regulated by the activity of metalloproteins, COX-1 and COX-2 and the reciprocal increase in expression of protective enzyme HO-1 observed in the gastric mucosa compromised by stress. Furthermore, the CO-induced gastroprotection against stress damage seems to be independent of NO, which is another important gaseous mediator. However, the mechanism of interaction among CO, NO and another potent gaseous molecule, H_2_S [64], in the gastric mucosal defense against stress requires confirmation in further studies.

## Figures and Tables

**Table 1:** Forward and reverse primers used in real-time PCR reaction to assess mRNA expression for β-actin, heme oxygenase (HO)-1, HO-2, hypoxia inducible factor 1α (HIF-1α), inducible nitric oxide synthase (iNOS), cyclooxygenase (COX)-2 and tumor necrosis factor α (TNF-α).

Gene	Forward Primer	Reverse Primer
*β-actin*	5′-GATCAAGATCATTGCTCCTCCTG-3′	5′-AGGGTGTAAAACGCAGCTCA-3′
*HO-1*	5′-GTCCCAGGATTTGTCCGAGG-3′	5′-GGAGGCCATCACCAGCTTAAA-3′
*HO-2*	5′-CCGGGCAGAAAATACCCAGT-3′	5′-ATCAGTGCTTCCTTCCGGTG-3′
*HIF-1α*	5′-ATCCATTTTCAGCTCAGGACAC-3′	5′-GGTAGGTTTCTGTAACTGGGTCTG-3′
*iNOS*	5′-TGGTGAGGGGACTGGACTTT-3′	5′-CTCCGTGGGGCTTGTAGTTG-3′
*COX-2*	5′-ATCAGAACCGCATTGCCTCT-3′	5′-GCCAGCAATCTGTCTGGTGA-3′
*TNF-α*	5′-TGGGCTCCCTCTCATCAGTT-3′	5′-TCCGCTTGGTGGTTTGCTAC-3′
